# Cases of Scirrhous Ovaria

**Published:** 1829-08

**Authors:** J. Leonard

**Affiliations:** Surgeon.


### SCIRRHOUS OVARIA.

*Cases of Scirrhous Ovaria.* By Mr. J. Leonard, Surgeon.

Cases of diseased ovaria are by no means of rare occurrence, and there is little in the detail of the majority of them interesting to the practitioner: they are generally well defined, and readily distinguishable from diseases affecting the other pelvic viscera; the cases running the same course, and terminating sooner or later fatally. Occasionally, however, we meet with a case worthy of selection, and such a one is the first of the two I shall narrate: it is remarkable for the rapidity of its progress, the size it attained, and from its being unaccompanied by those symptoms of debility, which are rarely absent whenever organic disease exists. Perhaps the age of the patient, being in the prime of life, and the short time she was a sufferer, account for the latter circumstance. The other case, although the diseased ovarium was of considerable size, is not very remarkable.

Case I.—Mrs. S , aged thirty-five, was delivered of a child fifteen months ago. The labour was protracted; the appearance of the abdomen had been remarked, during the progress of gestation, as being very unusual. The enlargement of the uterus with its contents went on naturally; but on the left side of the uterus was a tumor, about the size of a child's head at the time of birth. After the removal of the placenta, the contracted uterus was perceptible through the parietes of the abdomen, rather less than the tumor, which retained the same relative position, and was as firm to the touch. Her recovery was tedious; but, during the period of lactation, which continued seven months, her health was good, and she felt no inconvenience from the tumor, except a pain in the lumbar region occasionally, which readily went off. Soon after the infant was weaned, the pain in the lumbar region became more severe, and descended to the os sacrum. She described the pain as striking through to the left side of the lower part of the abdomen, down the thigh in the direction of the crural nerve, to the inner condyle of the os femoris, and up to the umbilicus. The tumor now seemed to occupy a more central situation in the abdomen, which had the appearance of pregnancy in the seventh month: (this was four months before her death.) There was no return of catamenia, nor any appearance of uterine discharge, till near the fatal termination of the disease. She was now much troubled with symptoms of uterine irritation, as sickness and vomiting, pain in the breasts, with reappearance of milk. The bowels were obliged to be regulated with mild laxatives.

In the early stage of the tumor, she had been ordered to take a combination of Pil. Hydrarg. and Antimon. Tart. as an alterative; but, not being attended with beneficial results, and having been pushed as far as circumstances would permit, it was discontinued. Venesection and leeches were frequently had recourse to, as the pressure on the large vessels occasioned a great determination of blood to the head; and two large caustic issues were opened in the lumbar region, and cicuta was prescribed. Nothing, however, had the least effect in checking the progress of the tumor: it continued to increase; the fits of sharp lancinating pain became more frequent, and lasted longer, accompanied with strong bearing-down pains, similar to labour, which were moderated by opiates. The functions of the bladder gradually became affected by the pressure, so that the male catheter was often required. Her size was much greater than that of a woman at the period of parturition; the integuments of the abdomen were extremely tense, and shining with patches of dark-coloured inflammation, threatening gangrene; and the pressure on the diaphragm impeded respiration.

She expired after a severe paroxysm of difficult respiration and vomiting, which lasted five days.

With four medical friends, I inspected the body twenty-four hours after death. The parietes of the abdomen were extremely thin, and the ensiform cartilage, and the cartilages of the lower ribs, were pushed out by the tumor, a small portion of the upper part of which was covered by the omentum. It proved to be the left ovarium, covered by its peritoneum: it was smooth and shining. Upon being cut into, it was found to be traversed by ligamentous bands, almost as hard as cartilage; the centre was rather softer; its artery was larger than the common iliac. It weighed sixteen pounds five ounces avoirdupois. The jejunum and ileum were pressed into the spaces on each side of the spine, and the liver was very small. The stomach was more vascular than common. The gall-bladder was completely filled with concretions, to the astonishing number of 108, one of which is as large as a nutmeg. These I have preserved. The other viscera were healthy, and there was very little appearance of œdema or serum in the cavity of the abdomen.

Case II.—Mrs. H., aged sixty-two, had complained of difficulty of breathing for a considerable time. Her belly was tumid, and fluctuation was evident. On the left side it was harder than on the right, and, upon pressure, a hard, irregular, knotty tumor was perceptible. Her legs were œdematous. As far as I could learn, the history of her case is shortly this: She had complained for years (perhaps sixteen or eighteen, to the best of her recollection,) of a very intense pain referred to the sacrum, shooting through to the pubis; latterly her stomach had been very irritable and her bowels irregular; and, about two months previous to the time I saw her, she first remarked the swelling of the abdomen, and that the secretion of urine was diminished.

Finding her in a state of extreme debility, and feeling satisfied that the ascites was only symptomatic of diseased ovarium, I gave an unfavorable prognosis. At the urgent desire of the sufferer, I drew off a quantity of fluid by tapping, which relieved the feeling of suffocation under which she laboured. She gradually sunk, and in ten days expired.

The left ovary was diseased in this case also. The tumor was of the true scirrhous character, irregular on its surface, intersected internally by ligamentous bands, and softer towards the centre, where a small irregular cavity contained about a teaspoonful of fetid sanies. It weighed four pounds two ounces. The right lobe of the liver was scirrhous in several places; and in the aorta were several patches of ossification.

*6, St. Martin's street, Leicester square.*

---

